# Adolescent Female Users’ Avatar Creation in Social Virtual Worlds: Opportunities and Challenges

**DOI:** 10.3390/bs14070539

**Published:** 2024-06-26

**Authors:** Chaeeun Ko, Seongcheol Kim

**Affiliations:** School of Media and Communication, Korea University, Seoul 02841, Republic of Korea; chd1237@korea.ac.kr

**Keywords:** social virtual world, social representation theory, avatar, adolescent, identity

## Abstract

Many adolescent females are active online, finding creative ways to express themselves through evolving social media technologies. Social virtual worlds (SVWs), distinguished by extensive avatar customization features, provide them with unique opportunities to craft virtual identities and explore diverse facets of self-presentation. This study investigates adolescent females’ construction of avatars in Zepeto, a South Korea-based global SVW platform. Employing social representation theory (SRT) as a theoretical framework, this study conducted in-depth interviews with adolescent female Zepeto users to uncover their perceptions of and motivations behind avatar creation in SVWs, as well as to explore how the interplay of virtual and real worlds presents them with various opportunities and challenges. While the prospects of SVWs remain uncertain, examining how these young users interpret and experience SVWs contributes to identifying potential strategies to enhance the sustainability of these platforms.

## 1. Introduction

With the shift in a large part of contemporary interpersonal communication to the digital space, online spaces have become a major socializing venue for adolescents [1,2], enough to be referred to as a second reality. For adolescents, who are undergoing an intensive identity formation yet have limited resources in reality, social media provides a platform to express themselves and interact with others in a way that transcends physical boundaries [3,4]. Particularly noteworthy is the active presence of adolescent females in online spaces. They dedicate more time to engaging in social media activities than their male counterparts, investing considerable effort into creating appealing online self-presentations [5,6]. In the USA, a higher percentage of females (48%) aged 13–17 reported being online almost constantly in their lives compared to males (43%) [7]. Similarly, in South Korea, girls spend an average of 217 min per day on mobile internet, exceeding boys’ usage by 37 min [8], which suggests that their online experiences constitute a considerable part of their lives.

Social virtual worlds (SVWs) provide adolescents with novel opportunities for self-representation via sophisticated avatars. With extensive customization features, users can create their avatars as desired, shaping them to closely resemble or diverge entirely from their actual selves. This capacity for creative self-reinvention sets SVWs apart from the more reality-bound representations common in social networking services (SNSs). While engaging with avatars in this immersive environment, adolescent users can experiment with their self-representation, form new relationships, and learn from their peers and other users. These experiences align with the developmental tasks commonly associated with adolescence [9].

Adolescents remain relatively underrepresented in the SVW literature, as previous studies predominantly focused on adult users. Adolescents constitute a significant proportion of SVW service users, which is evident in the popularity of platforms like Zepeto, where the largest user demographic consists of females aged between 13 and 21 [10]. A few studies such as Mäntymäki and Riemer [9] have contributed to an understanding of how adolescents perceive and engage with SVWs as digital habitats, but our knowledge regarding their avatar creation practices is still limited.

Kafai et al. [11] highlighted this research gap and identified the various resources, motivations, and constraints involved in tweens’ avatar creation in the Whyville SVW service. They suggested that virtual worlds offer a range of expressive resources that support fluid identity exploration among tween users, although the extent to which tweens could utilize these resources depended on their skills and status in the program [11]. During the timeframe of Kafai et al.’s study [11], platforms like Whyville offered two-dimensional avatars with low-resolution graphics; avatars only displayed the upper half of the body, and assembling facial features was a complex task. With the rapid development of visual technologies, the context of SVW avatar creation has changed significantly over the past decade. In contrast to the limited options of earlier platforms, emerging SVW platforms like Zepeto offer highly customizable avatar design systems, allowing users to manually adjust facial feature proportions and even leverage generative AI technology.

Furthermore, the current generation of adolescent users differs significantly from their counterparts of a decade ago. They are often considered digital natives, having grown up with technology during the early rise of SVWs in the 2010s. Consequently, it is reasonable to assume that both the agencies and resources influencing adolescent avatar creation have evolved considerably. These changes underscore the need for updated research in this area to capture the contemporary practices and experiences of adolescent users in SVWs.

This study investigates how adolescent female users construct identities through avatars in Zepeto, a South Korea-based global SVW platform, examining the interplay between virtual and real-life identities. Employing semi-structured interviews and the social representation theory (SRT) as a framework, it presents a social representations map of adolescent female users’ views on avatar creation in SVWs. By this, we seek to shed light on how they build virtual identities and cultures as well as the opportunities and challenges they encounter in that process. Given that choices made in crafting avatars reflect how the user interprets the context [12], examining these behaviors can provide insights into users’ overall perceptions of the SVW. This study comprehensively explores adolescent female users’ interrelated perceptions by analyzing the structural relationship of their social representations based on SRT.

This paper presents an overview of the prior research on adolescent females’ online self-presentation and SVW and avatar-based representations, then summarizes SRT as this study’s theoretical foundation. The process and findings of semi-structured interviews, content analysis, and core-periphery analysis are presented, articulating adolescent female users’ perception structure regarding SVW avatar creation in a three-section social representations map.

## 2. Literature Review and Theoretical Background

### 2.1. Online Self-Presentation of Adolescent Female Users

Identity formation is inherently shaped by social interactions, and social media, as a prominent platform for connecting with others, has become an integral part of adolescents’ identity development [13]. The act of self-presentation via digital personas is especially significant for those who are navigating a developmental phase characterized by active exploration and experimentation with their identities [14,15]. While participating in online environments, adolescent users develop self-perceptions and absorb societal norms through the process of observing and comparing themselves with peers [16].

Self-representation in the online sphere presents adolescents with both benefits and challenges, as outlined in Table 1. Online platforms have encouraged adolescents to engage in creative forms of self-expression and interaction that seamlessly blend text and images [17,18]. In terms of relationships, the absence of physical limitations in online environments enables individuals to expand their social circles beyond geographical boundaries and explore their interests with others who share their passions [19]. Research indicates adolescents are more likely to disclose their emotions, concerns, and vulnerabilities in computer-mediated communication settings due to reduced sensory and contextual cues; this enhances the quality of their friendships and eventually their well-being [20]. The online space is also often perceived as a relatively sheltered environment, allowing users to interact with their peers with limited adult supervision [9,17]. Virtual communities can serve as valuable “safe spaces” or “refuges” for users to express and explore identity issues, particularly for marginalized youth who frequently encounter hostility and discrimination in the physical world [19,21].

Adolescent females have been active users online, both as participants and content creators on social networking platforms [16]. One study reported adolescent girls dedicate more time to online activities involving smartphones and social media compared to boys, who tend to spend more time gaming [5]. Moreover, adolescent girls put greater effort into online self-presentation than boys, emphasizing the need to appear attractive on Facebook and Instagram [6]. In contrast to boys, girls often anticipate or even request their friends to like their posts as a way to enhance their image, a behavior that may be linked to real-world studies indicating that females place greater importance on committed friendships [6].

While digital technologies provide adolescent girls with novel platforms for self-expression and exploration, a noteworthy concern is that girls can internalize idealized beauty standards widely shared in the media, which may lead to more negative body image perceptions [22,23]. The meta-analysis conducted by Groesz et al. [24] indicates that the thin beauty ideal presented by the media has a notably more detrimental impact on body image among individuals under the age of 19. Adolescent girls also tend to engage in body comparisons most frequently with their peers and fashion models, and this tendency becomes more pronounced when they have internalized sociocultural ideals or when their body images are unstable [25]. On SNSs, likewise, as adolescent girls participate in self-presentation to garner peer recognition, the process of peer comparison can contribute to a self-perpetuating cycle of dissatisfaction and insecurity regarding their bodies and self-worth [16,23,26].

The capabilities to enhance or modify appearances afforded by social media can facilitate further reproduction and dissemination of this idealized notion of beauty. The high level of control afforded adolescent social media users allows them to selectively refine their self-images to more closely match their ideals [16]. Under pressure to constantly appear “perfect” in their online images, it is not uncommon for girls to edit their photos to portray an idealized version of themselves to be socially accepted and included [13]. Here, teenage girls learn and try to achieve peer standards of beauty, often featured by “flawless” skin and body, which are validated by peers through feedback mechanisms such as likes and follows on the platform [13,16]. Gendered norms of self-presentation seem to persist within digital spaces [6,13], potentially causing girls to shape their self-expression to conform to social expectations [27].

Adolescent girls’ online self-presentation entails various interpersonal risks as well. Inadequate internet literacy or limited platform affordances can lead to privacy risks on SNSs [17,18]. While the anonymity and minimized authority of online spaces often foster more intense personal disclosures (known as the *online disinhibition effect* [28]), these attributes can also increase the chances of interpersonal victimization such as cyberbullying or online harassment [20,29]. Alarmingly, 46% of US adolescents aged 13 to 17 reported having experienced cyberbullying at some point in their lives; of these, 31% believed they were targeted due to their physical appearance [30]. Cyberbullying was identified as a significant mediator of the connection between social media usage and mental well-being in girls, while this mediation was not observed in boys [31]. In addition, young online users face the risk of involuntary exposure to sexual content or unwanted sexual conversations [32]. Longobardi et al. [33] found adolescent females with body image concerns are more prone to engage in intimate relationships with strangers online, which consequently heightens their vulnerability to online sexual victimization. Given the emergence of immersive media that potentially brings new or expanded risks, it seems imperative to examine the opportunities and challenges associated with self-presentation on such platforms, including SVWs.

### 2.2. Social Virtual Worlds and Avatar Creation

A SVW is an immersive online environment that facilitates open-ended, collaborative interactions within virtual communities [34]. SVWs are frequently referred to as a type of “metaverse”, a term coined by Neal Stephenson in his novel *Snow Crash* to describe a virtual reality [35]. SVWs exemplified by platforms such as Second Life differ from game-based virtual worlds such as World of Warcraft in that they prioritize freedom of choice and open social interactions over predefined gameplay storylines [36,37]. Such social engagement enhances a sense of immersion within the SVWs [38]. Virtual communities within SVWs, characterized by interactivity, three-dimensionality, and real-time feedback [39], offer users the opportunity to engage in a diverse range of interactions, from role-playing in lifelike environments to trading virtual property rights using in-world currencies.

Adolescents have been active users of virtual worlds since their early iterations, seamlessly crossing the line between offline and virtual realms [9,40]. According to Metaversed [41], 51% of metaverse users are estimated to be aged 13 and younger; this figure rises to 78.7% when including users up to 16. The predominant user of the most popular metaverse services—Fortnite, Minecraft, and Roblox—is between 12 and 13 years old [41]. In the case of Zepeto, likewise, teenagers account for 80% of its user base [42]. However, research on this adolescent demographic in the context of virtual worlds has been relatively limited, partly because previous studies primarily focused on adult-oriented platforms like Second Life or World of Warcraft [9].

Table 2 shows different examples of virtual world services, including Zepeto, that are known to be popular among adolescents.

For adolescents, SVWs offer open environments, described as “identity playgrounds” by Kafai et al. [11] (p. 24), where they can experiment with self-presentation and social relationships while they navigate the path to adulthood. Adolescent users find enjoyment in escaping from real-life stress and engaging in social interactions within virtual communities, which Mäntymäki and Riemer [9] identified as a significant predictor of their intention to continue to use SVW. There are also notable concerns regarding the safety of young users who face potential harm such as sexual abuse, cyberbullying, or online grooming in SVWs that can hinder their participation in these worlds [40].

Research shows that female users of SVWs are primarily motivated by social interactions, role-playing, and activities like shopping and exploration [37,43]. Young users often engage in role-playing to explore identities different from their real-life personas [43], seeking entertainment in SVWs [37]. Zhou et al. [34] suggest that female users tend to value utilitarian and social benefits provided by SVWs, while male users derive more satisfaction from hedonic benefits. Females are also likely to spend more time interacting with empathizing objects, such as shopping or clothing options, compared to systemizing objects like blocks and buildings in SVWs [44]. These findings suggest that female users, particularly adolescents, engage with SVWs in distinctive ways, driven by their unique motivations and values.

In comparison to traditional SNSs, communication within SVWs exhibits two distinct features. First, in terms of constructing one’s digital persona, SVWs offer an almost limitless scope for crafting virtual 3D avatars [45]. Unlike SNSs that often serve as extensions of users’ offline existence by promoting reduced anonymity through personal profiles, SVWs invite users to embody desired personas or adopt fictional characters detached from their offline identities [16]. Highlighting the rich affordance of the SVW platforms, Kaplan and Haenlein [45] describe SVWs as a category of social media that provides a higher level of social presence and self-presentation opportunities than other types by replicating multiple dimensions of face-to-face interactions without restrictive rules.

Second, in the context of cultivating social connections, SVWs can offer users greater prospects for establishing new relationships based on synchronicity. In contrast to SNSs that are known for primarily supporting interactions within pre-existing social circles [46], SVWs are more conducive to serendipitous discoveries and chance encounters by their inherent design. While concerns have arisen about “filter bubbles” on SNSs and other algorithm-driven media platforms that lead to biased exposure to users with similar viewpoints [47,48], interactions within SVWs are fundamentally shaped by time and space synchrony. Platform users who coincidentally venture into the same virtual location at the same time can partake in shared moments and spaces together. This kind of relationship-building may encourage users to invest more effort in crafting avatars, potentially with less pressure to maintain a consistent self-concept [49], as avatars become the sole means of representing themselves among their new acquaintances in the virtual realm.

Avatars, defined as “general graphic representations that are personified by means of computer technology” [50] (p. 20), play a crucial role in self-expression and social interaction in SVWs. Advanced graphic technologies and the absence of physical limitations allow individuals creative freedom in crafting their digital representatives. The highly customizable nature of avatars provides users the ability to adjust their avatars’ appearances according to personal preferences, albeit within the constraints of available options and resources [27]. Given that the process of creating an avatar involves a series of choices that reflect users’ personal desires and perceptions [36,51], examining user thoughts about avatar creation can offer valuable insights about users’ interaction preferences within these immersive digital environments.

Previous research supports the view that avatar creation goes beyond mere visual customization, playing a pivotal role in shaping individuals’ engagement within SVWs. Yee and Bailenson [52] introduced the term, “Proteus Effect”, positing appearances of avatars significantly shape users’ self-perceptions and social interactions within the virtual world. Research findings showed that participants assigned to more attractive avatars were more willing to disclose personal information, while those who had tall avatars showed higher levels of confidence [52,53]. Other studies have found that the relationship between users and their avatars significantly influences user behavior. According to Hooi and Cho [54], users of Second Life who reported that their avatars resembled their real selves were more likely to perceive an alignment of thoughts and values between themselves and their avatars. This sense of homophily had a positive impact on self-disclosure, driven by increased self-awareness and a stronger sense of self-presence [54]. Similarly, Takano and Taka [55] found that strong user identification with avatars, enhanced through customization, fostered a sense of group belonging within a Japanese virtual community, Pigg Party. Relation expansion was facilitated through practices like self-disclosure and self-presentation, which in turn encouraged users to spend more time in virtual conversations [55].

The flexible nature of avatar construction produces varied outcomes based on users’ choices and preferences. While advanced technologies such as facial recognition and body scanning have enabled digital representations that highly resemble users’ actual appearances [56,57], users can choose to create a completely different avatar that mirrors their ideal self. Research findings regarding whether individuals tend to design idealized or realistic avatars have been inconclusive. For instance, Ducheneaut et al. [36] observed users creating avatars that deviate from their real-life attributes in terms of increased attractiveness, physical fitness, and distinctiveness from the crowd. In contrast, Zimmermann et al. [58] discovered a high level of congruence between avatar, actual self, and ideal self concerning physical and demographic characteristics (e.g., height, weight, age, gender) across varying online activity contexts. Although users commonly enhance physical attributes that they are dissatisfied with in real life, as Messenger et al. [49] proposed, they balance the motives of self-verification and self-enhancement to compose avatars that blend actual and enhanced versions of self. Villani et al. [59] also found that adolescents perceived their avatars as equally resembling both their actual and ideal selves. According to Bimberg et al. [60], avatar design behaviors are also influenced by the specific settings of virtual environments. Higher feelings of presence in the virtual environment are associated with a greater tendency to create avatars that reflect users’ actual selves [60]. Additionally, low diversity in representations can restrain minorities from expressing their identities through avatars [61].

The extent of the discrepancy between actual self and avatar self also hinged on specific identity traits. Nowak and Rauh [12] found that users preferred human (i.e., anthropomorphic) avatars consistent with their own gender. In a study conducted by Sung et al. [62], participants demonstrated matches of 98.5% for gender, 87.1% for ethnicity, 62.9% for age, and 51% for occupation when compared with their avatars in online social contexts. Even though many virtual world users reported having multiple avatars [63], they tend to maintain consistency for core identity traits such as gender and race, while modifying more peripheral traits such as hair, face, and clothing (*a hierarchy of physical variation* [49]).

Patterns of avatar creation were also shown to differ depending on users’ demographics. Ducheneaut et al. [36] found males tended to make avatars that draw attention, whereas females were more inclined to create idealized avatars. Older users typically opted for avatars resembling idealized versions of themselves, while younger users tended to follow trends in avatar creation [36]. Kafai et al. [11] also identified trends as a motivation for tweens’ avatar creation, with tweens either aligning with or against the trends. Other primary motivations highlighted by Kafai et al. [11] include pursuing aesthetic preferences, mirroring aspects of their real selves (e.g., physical appearance, personal likes, affiliation, desires unfulfilled in real life), and functional purposes such as disguise. Villani et al. [59] also noted that adolescents’ avatar creation varies based on their age and sex; older adolescents tended to incorporate more facial details and body features into their avatars. Adolescent females tended to create avatars with more clothing and ornamentation, while males often added sexual features to their avatars’ faces, bodies, and clothing [59]. Avatar preferences can also vary across cultures, as different cultures favor certain traits (e.g., cuteness) to different extents [64].

Earlier research indicates that real-world societal norms also influence how avatars are created and perceived. Martey and Consalvo [65] examined the avatar appearance of Second Life users, revealing that users often crafted avatars that aligned with the societal norms shared by the individuals they engaged with. For instance, a female avatar with light skin color wearing accessories and dressed in revealing attire tended to be perceived as more likable by the community. Mills [39] also found virtual world reflections of the predominant female beauty standards, with avatars reinforcing Western ideals that favor light skin tones and a body size that falls between not-too-thin and not-overweight. Morrison [27] notes that girls often perceive the imagined gaze judging their appearance both online and offline. This perception places pressure on them to craft an idealized avatar by removing imperfections as a practice of commercially influenced versions of girlhood [27]. A limited diversity in avatar options can further strengthen stereotypes and biased interactions [66]. Thus, even though it may seem that users have complete creative freedom to shape their avatars, socially constructed beauty norms may impose constraints on avatar creation practices within SVWs [39].

Although previous studies have contributed to the understanding of avatar creation behaviors and their implications among SVW users since the late 2000s [11,27,36], the emergence of technologically advanced SVW platforms such as Zepeto, which allow for more sophisticated avatar customization, necessitates updated research on this subject. In particular, the exploration of adolescent females who have actively engaged in online culture, including SVWs, to shape and present their identities is a promising research area. As a prominent user segment, this demographic holds the potential to provide valuable insights into the future landscape of SVWs. This study focuses on avatar creation among female adolescent Zepeto users, along with their general perceptions of the SVW, and discusses challenges and opportunities these users encounter, drawing from an analysis of the social representations that mirror their collective thoughts and practices.

### 2.3. Theoretical Framework: Social Representation Theory

Social representation theory (SRT) is a theoretical framework that has been extensively employed in social science research to explore how individuals and communities collectively understand and construct knowledge about various social objects. It underscores the idea that people, as members of social groups that share similar experiences and characteristics, build common knowledge via ongoing community interactions [67,68]. This common knowledge is presented as social representations, which are composed of two layers of elements: cores and peripherals [69]. While the cores represent more stable elements of the social representations, peripherals are more dynamic and variable, allowing social representations flexibility to fit a variety of contexts [69].

SRT is particularly well-suited for investigating perceptions related to novel or innovative social objects, which renders it a useful approach for studying views of SVWs. Jung and Pawlowski [70] explored the social representations of virtual entrepreneurship, focusing on the novel business opportunities afforded by SVWs. The applicability of SRT in the context of SVWs is further supported by Park and Kim [71], who used it to explore how people with disabilities construct virtual identities through avatars. SRT has also proven effective in exploratory studies that addressed other emerging technologies; for instance, Choi et al. [72] employed SRT to examine understandings of fintech among industry stakeholders, and Jang et al. [73] investigated managers’ perceptions of chatbot use in the financial sector. In each of these studies, SRT provided a valuable framework for understanding how specific social groups make sense of new social objects.

Another rationale for employing SRT in this study is its effectiveness in identifying collective views within specific demographic groups. For example, Ahn and Jung [67] applied SRT to compare the social representations of smartphone addiction between digital natives (DN) and digital immigrants (DI), highlighting generational differences in how the phenomenon is understood. SRT has been employed in studies that specifically focused on adolescents, identifying their social representations on various topics such as smoking [74,75], body image [76], elderly people [77], adaptation of immigrants [78], AIDS [79], and climate change [80]. Using the SRT framework, these studies provide insights into shared knowledge, attitudes, and perceptions among adolescents who are engaged in similar developmental tasks and interests. The current study extends this line of inquiry to examine adolescents’ perceptions related to SVW avatar creation.

SRT offers the advantage of analyzing the structure of social representations, which comprises core and peripheral elements [69]. While previous studies have suggested inconsistent patterns in users’ avatar creation depending on motivations or specific identity traits [11,36,49,62], SRT provides a framework to uncover potentially divergent structures of perceptions regarding avatars and assess their respective significance. Additionally, as avatar creation involves not only users’ personal preferences but also their interpretations of the SVW context, including how it differs from reality or typical games, SRT is a useful approach to analyze the interconnected perceptions and experiences of users and explore how these perceptions relate to, compete with, and support one another. In summary, SRT is highly applicable to the investigation of how adolescent females collectively perceive and construct avatars, especially within the dynamic context of the SVW, which represents a relatively recent and pioneering social concept.

Given this background, specific research questions of this study were established as follows:

**RQ1.** How do adolescent female users construct their avatar identities, including traits such as age, gender, occupation, skin color, and body shape, in social virtual worlds?
**RQ2.** How do adolescent female users perceive social virtual worlds in comparison to reality and games?
**RQ3.** What are the opportunities and challenges experienced by adolescent female users in social virtual worlds?

By applying SRT to these research questions, this study aims to uncover the underlying structures of social representations related to adolescent females’ avatar creation and experiences in SVWs. Specifically, identifying the core and peripheral elements of these social representations will help to elucidate the drivers and patterns that shape their avatar creation practices and engagement within SVWs, illuminating the opportunities and challenges involved in those experiences.

## 3. Methodology

### 3.1. Research Context

Zepeto, the research setting for this paper, is a SVW service run by Naver Z, a subsidiary of Korean tech giant Naver. Since its launch in 2018, Zepeto has grown into a global metaverse platform with over 400 million users as of April 2023 [81]. Naver Z describes Zepeto as a “universe where your imagination comes true”, allowing users to interact globally through unique avatars and create virtual items and backgrounds [82]. Notably, young female users make up a substantial portion of Zepeto’s user base; roughly 70% of its monthly active users are estimated to be females, with the majority falling between the ages of 13 and 21 [10]. Another analysis indicates Zepeto’s primary user demographic consists of girls in their mid to late teens who are oriented toward consumption and communication [42].

Unlike typical online games, Zepeto users create their own experiences in themed spaces called Worlds, which replicate real-world locations like school classrooms and Han River Park in Seoul. Users can take photos of their avatars and post them on their feeds, similar to traditional social media. Avatars are central to engagements within Zepeto, with over a billion virtual fashion items available for customization [83]. Partnering with fashion brands like Gucci and Nike, and K-pop artists like BlackPink [83], Zepeto offers users the chance to extend their real lives or explore new experiences virtually.

Zepeto’s functionalities provide sophisticated control in avatar creation. Zepeto Studio, launched in 2020, allows users to sell their designs, expanding avatar options. The Custom PRO function, introduced in November 2022, enables detailed customization of avatars by manually adjusting specific figures of the face. Customization requires virtual currency, Coins and ZEMs, with Coins earned through quests or advertisements and ZEMs typically purchased with real money at the exchange rate of 14 ZEMs to one dollar. ZEM items mostly cost 1–10 ZEMs each, though creators can price their products between 0 and 100 ZEMs.

### 3.2. Semi-Structured Interviews

Data were collected through in-depth interviews carried out within the Zepeto platform using its internal messaging function. To recruit participants, the researcher initiated contact by sending interview requests to users encountered in Zepeto Worlds. The primary focus was on those users who had disclosed their ages in their profiles, and they were initially asked to confirm whether they were adolescent females before proceeding with the interview. A semi-structured interview questionnaire had been prepared in advance, though the questions were adjusted and reformulated based on the natural flow of conversation with the interviewees. Each interview had an approximate duration of one hour.

The interview commenced by inquiring about the participants’ overall perception of the Zepeto world, seeking to understand their perspective on questions such as “What kind of world is Zepeto for you?” or “What is your biggest reason for using Zepeto?” Subsequently, the discussion shifted towards the perception of avatar creation and the motivations behind it. Participants were asked whether they preferred to craft avatars that mirrored their own characteristics or diverged from them. Detailed aspects of avatars, including age, gender, occupation, skin color, and body shape, were explored individually. Finally, participants were queried about their basic Zepeto usage information, including the duration of usage, average frequency of use, and the time and real-world money invested in avatar creation.

A total of 15 Korean participants, all of whom were early adolescent female Zepeto users aged between 9 and 15, were interviewed. A summary of the frequency analysis concerning interviewee characteristics is provided in Table 3. The majority of participants (11 individuals) indicated that they use Zepeto almost daily, enabling an exploration of the everyday experiences of adolescent females. The participants’ usage durations spanned from as short as 10 days for one who had recently joined the platform to as long as 5 years for one who had been on the platform since its inception. Most of the adolescents interviewed had been using Zepeto for more than a year and were familiar with the avatar creation process and the Zepeto environment. If younger participants were unclear about a question during the interview process, the interviewer would rephrase the question in plain language and proceed step-by-step to ensure comprehension. This approach helped to mitigate any difficulties young participants might have had with the more abstract questions.

### 3.3. Content Analysis

For content analysis, the interview transcripts were broken into sentences to distinguish sampling and coding units. After thoroughly reviewing the entire text, we employed relevance sampling to select the sentences pertinent to our research questions [84]. These textual units were then analyzed based on an open coding process [70,71]. Initially, the first coder extracted 39 codes. The second coder independently coded the data, referencing the initial codes. During discussions between the coders, topics were restructured and refined, eliminating those with unclear or overlapping meanings, resulting in a final set of 17 codes. Using these finalized codes, both coders independently assigned 2–3 topics to each sentence extracted from the transcripts. The interrater reliability, as measured by Cohen’s Kappa, was found to be 0.91, signifying a high level of agreement. The final topics, along with illustrative examples, are presented in Table 4.

### 3.4. Analysis of the Structure: Core and Periphery Analysis

To analyze the structure of the topics derived from the content analysis, core and periphery analysis was conducted using the statistical software UCINET, following the core-periphery algorithm devised by Borgatti and Everett [85]. First, a matrix of co-occurrence among topics was generated, and each topic’s coreness score was computed with UCINET. This led to the categorization of topics into two distinct groups, either core or periphery; 6 topics were identified as core elements, while the remaining 11 topics were classified as peripheral elements (Table 5).

### 3.5. Mapping Social Representations

**Figure 1:**
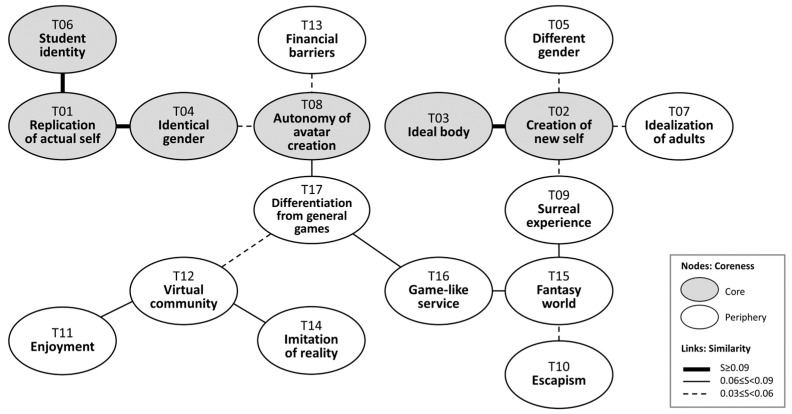
Social representations map.

Based on the core-periphery analysis results, a social representations map, depicted in Figure 1, was generated using the maximum tree method [86]. Beginning with the code with the highest coreness score, the map was expanded sequentially by connecting a code that had the highest similarity score to any existing code but had not yet been included in the map. The map serves as a visual representation of the interconnections among topics [87]. Each node within the map represents a specific topic, and the lines connecting the nodes illustrate the associations between these topics. Nodes are color-coded to indicate their core or periphery status, while the lines vary in style to signify the degree of co-occurrence between the two codes.

## 4. Results

The interviews revealed detailed insights into perceptions around avatar creation and experiences in SVWs among adolescent female users. Participants described a series of decisions they made for each component of their avatars, including facial details, hair, body shape, skin tone, clothing, accessories, and makeup. The time and money spent on avatar design varied, as shown in Table 3, with most participants spending less than an hour and little to no money, while a few invested significantly more time and resources. Participants displayed two major patterns in constructing avatar identities: sometimes they replicated their actual traits, and other times they adopted options to create virtual selves different from their real-life persona. These patterns reflect the diverse motivations that shape avatar creation among adolescent females, ranging from exploring new identities to expressing facets of their real selves in SVWs.

### 4.1. Replication of Actual Self in Avatar Creation (RQ1)

Replication of actual self (T01) exhibited the highest coreness score among the 17 topics, implying adolescent female users want their avatars’ appearances to resemble the users’ actual appearances. This tendency was often associated with the users’ satisfaction with the identity traits they possessed in real life. Most of the interviewees expressed a desire to designate their avatars’ occupation as “student”, akin to their own status (T06), mainly because they were content being students. Some of them mentioned the burden of being an adult, associating it with the duty of going to work. Additionally, some participants opted to remain as teenage students in the virtual world because it offered a sense of comfort and familiarity that mirrored their real-life roles.

The feeling of comfort with self-like avatars was applicable to gender as well, constituting a major motivation for creating avatars with identical gender (T04). Most of the interviewees felt more comfortable with operating avatars that shared the same gender identity as their real selves. Adopting a different gender for their avatars sometimes led to feelings of confusion or a sense of misrepresentation among users. As expressed by interviewees, statements such as “male or nonbinary bodies just do not fit me” and “it is not the real me if it is a different gender” highlighted this discomfort with gender incongruence in avatar representation.

**Figure 2:**
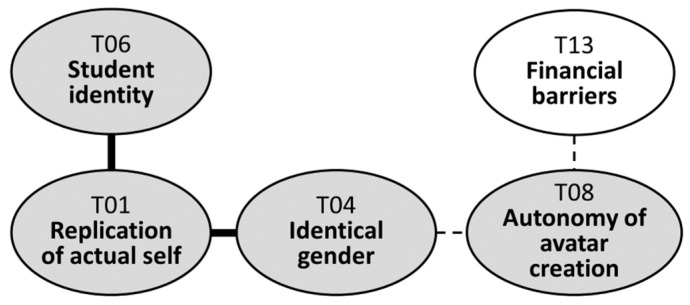
Replication of actual self in avatar creation.

Another motivation underlying the preference for same-gender avatars was that “you can vicariously satisfy yourself by putting on various styles you wanted to wear in real life”. This motivation was closely linked to the autonomy of avatar creation afforded by Zepeto platform (T08). Given the freedom to customize their avatars, users could fully leverage Zepeto as a space for self-expression, extending their identities into the virtual world through avatars described as “a Zepeto version of me” or “another me in Zepeto”. Yet, financial constraints (T13) faced during the avatar creation process could hinder the realization of this autonomy. Further elaboration on this topic will be provided in a subsequent section. Figure 2 shows the relevant portion of the map.

### 4.2. Creation of New Self in Avatar Creation (RQ1)

Adolescent female users also expressed a willingness to create avatars that differed from their real-world appearances (T02). One prevalent trend was customizing avatars to embody an idealized image of the body (T03), often stemming from users’ dissatisfaction with their actual appearance. For instance, several interviewees preferred to give their avatars lighter skin tones than their own, stating comments like “I feel like my current skin is too tan” or “I wish my skin tone were brighter [in the real world]”. This trend extended to body shapes as well, with adolescent females designing avatars with different weights and heights from their real bodies. Interestingly, body shape preferences were not uniform; those who perceived themselves as small or skinny wanted their avatars to be larger and taller, while others who viewed themselves as overweight desired smaller and thinner avatars. This implies a connection between real-world dissatisfaction and the creation of idealized avatars in the SVW, aligning with the findings of Messinger et al. [49] who observed that people tend to enhance physical traits they are less satisfied with in real life when creating avatars. Some interviewees said their avatars were more like their creation and felt separate from themselves because the avatar looked completely different from their own appearance.

**Figure 3:**
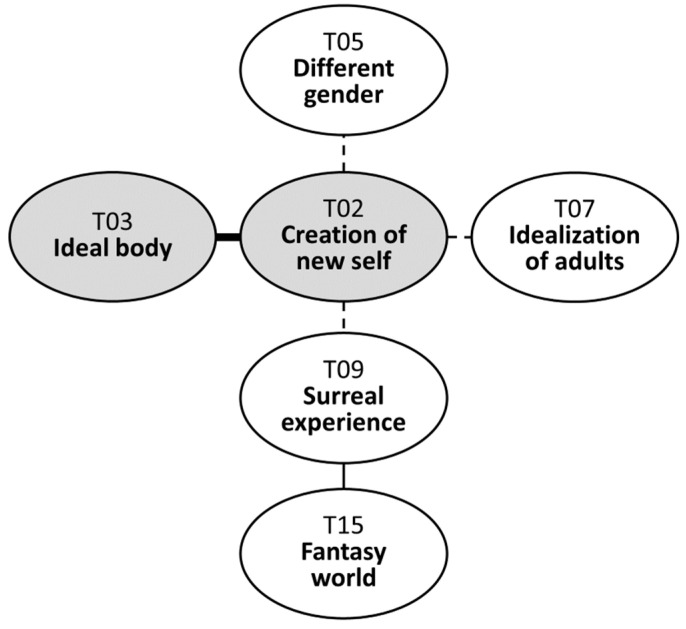
Creation of new self in avatar creation.

Physical appearance ideals are reflected in avatar creation precisely because the ideal is easily attainable in the virtual world. Zepeto allows users to engage in activities they would not be able to experience in reality (T09). As one interviewee mentioned, “You cannot change your height in reality” even though you “think a shorter height looks cuter” or vice versa. Another example would be age; although most of the interviewees chose to make their avatars look similar in age to themselves, a few of them preferred to create avatars that appeared older. This tendency was shown with an idealization of adults (T07), as they noted that “people in their 20s seem to enjoy more freedom with their lives”. A few participants also expressed interest in trying out a male gender avatar (T05), since the virtual world allows users to embody their imagination (“I have wondered how it would feel to be a boy and hang out with other boys”). The functionalities of virtuality, which enable users to change the height, skin color, gender, or age with just one click, seem to encourage adolescents to experiment with appearances that differ from their real-life selves. This reinforces the users’ perception that Zepeto is a fantasy world distinct from reality (T15). The relevant section of the map is depicted in Figure 3.

In summary, to address Research Question 1, it was found that adolescent female users customized their avatars’ appearances in varied ways, either consistent or inconsistent with their actual selves, driven by different motivations. Most users created avatars that mirrored aspects of their real selves, such as age and gender, because they were content with their real-life selves, found it more comfortable to operate avatars resembling themselves, or identified avatars with their actual selves. However, it was common for users to modify aspects they were not satisfied with, primarily skin color and body shape. A couple of users expressed curiosity about exploring alternative identities that were inaccessible in reality, leveraging the creative freedom and technological features available in Zepeto.

### 4.3. Adolescent Female Users’ Perception of the Social Virtual World (RQ2)

Addressing Research Question 2 involves exploring adolescents’ perception of the SVW in a broader context: how do adolescent female users distinguish the SVW from conventional games or reality, and what does the SVW mean to them? The interviewees perceived SVW platforms like Zepeto as distinct from typical games (T17) primarily in two key aspects: freedom of choice in designing avatars (T08) and ways of communication with others (T12). Unlike conventional games, which often feature preset characters that “cannot be customized in detail”, SVW platforms offer a wide array of choices for users to autonomously tailor their avatars. With these avatars serving as their digital representatives, adolescent female users engage in communication with others through various means such as chat, posts, comments, and even live streaming, which further sets Zepeto apart from general gaming experiences.

“I believe Zepeto is somewhat different from regular games”, one interviewee described; “the ability to share posts or host live broadcasts within the game platform felt incredibly novel to me”. The majority of the interviewees expressed satisfaction in forming new friendships through Zepeto, with some even suggesting that “meeting friends on Zepeto is better than real-life friendships”, since they can “share their emotions and concerns more comfortably” in the virtual world. Previous research has suggested that adolescents often find it easier to disclose their feelings in computer-mediated communication contexts, which boosts relationship quality [20]. Understandably, participation in such virtual communities brought enjoyment to adolescent users (T11), which users noted as a primary motivation for using Zepeto.

For users, Zepeto’s interactive elements encouraged them to perceive a resemblance between the SVW and the real world (T14). One interviewee expressed it as, “At times, it just feels like reality presented in a different format”. Interviewees reported background settings that resemble real-world places, such as Han River Park or a school cafeteria, further enhanced the sense of actually being in these locations. This phenomenon can be attributed to the concept of spatial presence, which broadly refers to “the feeling of being physically located in the virtual environment” [88] (p. 4). Users also noted that 3D avatars that closely resemble human bodies, complete with “sophisticated and realistic fashion items” and “intricate movements” supported by advanced graphic technologies, contributed to a perception of the SVW as realistic.

**Figure 4:**
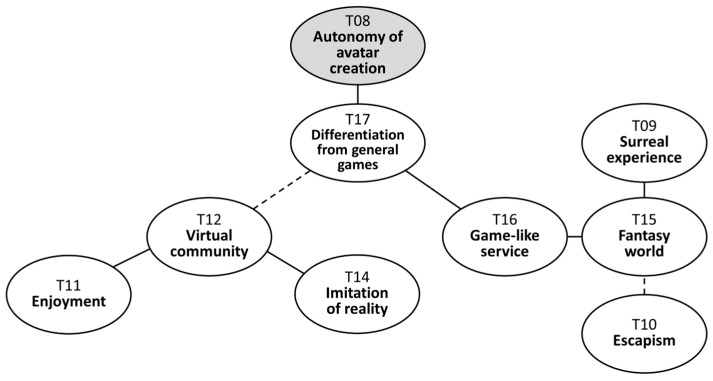
Adolescent female users’ perception of the social virtual world.

However, it is worth noting that Zepeto was still commonly perceived as a form of game (T16). One interviewee characterized the metaverse as “a casual virtual space that gives off feelings like playing games”. Others, albeit unconsciously, used the term “game” when referring to Zepeto during the interviews, such as stating “Zepeto is the funniest game”. This perception is closely linked to the idea that Zepeto exists in a fantasy realm distinct from the real world (T15). Due to the surreal capability to swiftly customize or alter one’s identity (T09), adolescents viewed Zepeto as a fantasy, which led them to perceive it as a game as well. This observation reveals an intriguing tendency among participants to equate fantasy with games; Zepeto is viewed as a game because it is not reality. This implies that, for adolescent females who participated in this study, it could be hard to imagine a fantasy world that does not belong to the realm of games. Some also highlighted the freedom of mobility, as they could teleport to various locations within the SVW, unlike in the real world. This autonomy and element of fantasy allowed adolescents to temporarily escape from the everyday pressures of real life and alleviate the stress associated with their schoolwork (T10). The corresponding map section is presented in Figure 4.

### 4.4. Opportunities and Challenges in the Social Virtual World (RQ3)

Our research findings highlight constraints and challenges adolescent females encounter while engaging in the avatar creation process in SVWs. One of them is financial barriers (T13) to the unconstrained acquisition of virtual items. Several interviewees commented that it was hard to purchase an adequate amount of items or virtual currency (e.g., ZEM in Zepeto, Robux in Roblox) for customizing their avatars precisely as desired. This limitation sometimes stemmed from parental disapproval or simply a lack of money in real life. As one interviewee explained, “My mom never allows me to spend money on games”. Notably, the perception of Zepeto as a type of game seems intertwined with this financial challenge, as it deterred parents from allowing their children to spend money on it based on a negative image of gaming.

The financial constraints adolescent users face in Zepeto present obstacles to their creating avatars that either differ from their actual selves (“I want to create another avatar that looks different from me, but I can’t afford to do so”) or imitate their actual selves (“I want to shorten my avatar height to resemble myself, but I can’t due to the expense of purchasing an appropriate body shape”). When asked about specific improvements to avatar customization functions on Zepeto, most participants expressed satisfaction with the current variety of options but desired cheaper items for purchase. These financial challenges exemplify the practical challenges that adolescent female users experience when they craft their virtual personas.

Another more implicit influence that constrains the avatar creation process for adolescent females is the idealized concept of beauty (T03). One interviewee offered the prevalence of “lookism” in South Korean society as a reason for wanting to give her avatar lighter skin, explaining that in Korea, a fair skin tone has been consistently preferred and associated with “attractive appearances”. Another interviewee observed that the default avatar options offered by the platform—a greater selection of slim body shapes compared to plus-size alternatives—seem to inherently exhibit bias. This aligns with the suggestion by Mills [39] who conducted a content analysis of the portrayal of female avatars on Second Life Marketplace. According to Mills [39], although users seem to have full control of avatar creation, it is gatekeepers who determine the boundaries of choices available for crafting avatars. Our analysis suggests that socially constructed ideals in the real world can naturally translate to virtual spaces through the avatar creation process, which relies on the platform’s affordances and user perceptions. This raises a thought provoking and somewhat concerning question about the reproduction of social norms within the virtual world.

Our findings indicate that adolescent female users also leverage the opportunities that SVWs offer in terms of identity extension, exploration, and detachment from the constraints of reality. In SVWs, our participants extended their real-world identities into the virtual realm, mirroring the traits and roles they are satisfied with or that constitute a significant part of their identity. For example, many participants wanted to portray themselves as students or females in SVWs, aligning their virtual selves with aspects of their actual identities that they felt comfortable with or wanted to represent. Projecting their real-world attributes, adolescent females extend their experiences to the virtual world via avatars that mirror their own characteristics.

SVWs provide a unique platform for identity exploration and social interaction that transcends the limitations of the physical world. Participants expressed the joy of vicarious satisfaction by experimenting with styles and personas that might be challenging or impossible to adopt in their daily lives, such as dressing up as idols or exploring different gender identities. The absence of physical constraints in SVWs enables adolescents to experiment with self-representation, which is an important part of developing toward adulthood [9]. SVWs also provide adolescents enjoyable opportunities to broaden their social circles and engage in virtual communities beyond geographical or algorithmic boundaries.

Such exploration sometimes took the form of engaging in fantasies that offered respite from the dissatisfactions of reality. Adolescent female users created avatars with idealized appearances, including alterations to body shapes and skin colors that contrasted with their real-life attributes. Participants also described using SVWs as an escape from the stresses of academic responsibilities, highlighting the role of these virtual spaces as fantasy worlds that provide relief and relaxation.

Contrary to our expectations, safety concerns did not emerge as a major theme in our interviews with adolescent female users. Instead, these young users expressed their enjoyment of engaging in virtual communities. They shared their experience of forming friend groups and exchanging virtual gifts on Zepeto. However, it remains crucial to ensure secure communication settings for adolescents, not only for their safety but also for their continued presence on the platform. For instance, one interviewee who identified herself as a user of both Zepeto and Roblox stated that she “used Zepeto more frequently due to the prevalence of abusive language in Roblox”. Another interviewee mentioned meeting other Zepeto users in person, indicating a latent interpersonal risk, even though the interviewees did not explicitly characterize it as a risk.

In summary, SVWs offer adolescent female users the opportunity to extend, explore, and temporarily detach from their real-life identities. These experiences enrich their engagement with SVWs and contribute to the complexity of their relationship with these virtual environments. As a parallel to self-expression in the real world, constructing avatars in SVWs is a social behavior that often demands resources. Consequently, an individual’s socioeconomic status and societal expectations shape their avatar creation behavior, which is interconnected with real-world resources and norms. Analyzing these opportunities and challenges sheds light on the interplay between reality and virtuality in the adolescent females’ SVW experiences.

## 5. Discussion and Conclusions

While SVWs have gained popularity among youth, research has primarily focused on adult users, leaving a gap in understanding adolescents’ perceptions and engagement. Adolescent females are active online users who leverage evolving media technologies for creative expression and social interaction. SVWs offer them unique opportunities to construct virtual identities and explore diverse facets of self-expression. Through interviews with adolescent female Zepeto users and analysis based on SRT, this study highlights their perceptions and motivations behind avatar creation and identifies how the interaction between the virtual and real worlds presents them with various possibilities and challenges.

The social representations map from the interviews reveals 6 core and 11 periphery topics. Replication of actual selves had the highest coreness score, but there was also a desire to create avatars that diverged from users’ actual selves. Most participants maintained their female gender and student status, though some explored different genders or occupations out of curiosity or idealization. Physical attributes such as body shapes and skin tones were often enhanced to align with idealized beauty standards or address real-life body dissatisfaction, expanding upon Messinger et al.’s [49] observation that user identity traits are represented to varying degrees in avatars.

Participants perceived Zepeto as a form of gaming due to its unrealistic functionalities, but its communication features differentiated it from other games. Adolescents, accustomed to socializing through SNSs, found Zepeto’s voice chat and video post functions familiar and enjoyable. In this sense, enhancing the sense of reality in interactions may help SVWs be viewed as “worlds” beyond games. Future SVW developments should focus on seamlessly integrating real-life interactive activities and establishing robust safety guidelines and policies to foster active user engagement.

SVWs provide significant autonomy in avatar creation, allowing adolescent females to embody identities unattainable in the real world. As the users blended their actual selves with experimental identities and styles, they explored the possibilities of self-presentation via avatars. However, although adolescent users generally perceived there were abundant customizing options, financial limitations restricted their ability to fully customize avatars to resemble themselves or explore different styles.

The results suggest that the real-world beauty standards can be reflected in the avatar creation process, potentially leading to a lack of diversity within the virtual world. For instance, if most users create avatars with fair skin, white individuals may be overrepresented in SVWs, while people of color are underrepresented. This can extend to other identity aspects like body shape, gender, and age. A lack of diverse representations in virtual worlds can hinder minorities from expressing their true identities through avatars [61], creating a space that perpetuates stereotypes of the “ideal” or “normal” body.

It is worthwhile to consider the long-term psychological effects of avatar creation on adolescents. Adolescents are in a critical developmental stage where they are exploring and forming their identities. On the positive side, SVWs can provide an enjoyable space for adolescents to experiment with different aspects of their identities. However, the pursuit of idealized virtual selves can exacerbate issues related to body image and self-worth, especially if avatars conform to unrealistic beauty standards. In the process of creating ideally attractive avatars, they may not only learn but also unwittingly reproduce real-world beauty norms, which can influence their self-perception and how they view others in real life.

These findings carry practical implications for enhancing the social and financial sustainability of SVWs. For SVW service providers, it underscores the need to promote diversity in product design. While adolescent female users now have the technical capability to create avatars in nearly any form they desire, they are still influenced by real-world norms when judging their appearances. This constraint limits the range of avatars they create [39] and poses challenges to the idea that adolescents can use virtual communities as a safe alternative space for self-expression [19,21]. SVW services should offer a wider range of body shapes and identity options, enhancing user identification with avatars and promoting diversity [54,55].

At a more fundamental level, reflecting the values of diversity and inclusion in SVW marketing is essential. This could involve diversity campaigns within the platform and featuring diverse avatars in promotional materials. As exemplified by the body positivity movement in the real world, showcasing virtual influencers with diverse traits can challenge idealized beauty standards. Marketing materials disseminated by Zepeto, whether within its platform, on its website, through blogs, or external media, should include avatars with diverse skin colors, body shapes, disabilities, and gender expressions to convey an inclusive vision.

When diverse customizing options are offered, ensuring their accessibility for adolescent users is vital. Our findings imply that financial barriers often prevent them from acquiring virtual items. Expanding alternative business models that allow young users to earn items through non-monetary means, such as watching advertisements, can make these options more accessible. Additionally, providing training for item creation and inviting more adolescents to participate in the virtual economy as sellers can effectively empower them to increase their resources while acquiring entrepreneurship experience [70].

Parents can make adolescents’ avatar creation experience more positive and healthier by understanding, discussing, and joining their children’s SVW activities. While appreciating the importance that virtual lives may have for their children, parents can discuss the content and interactions encountered in the virtual world, helping them process these experiences more constructively. Encouraging open communication about any feelings or concerns associated with avatar creation can help adolescents critically assess the influence of virtual interactions on their self-perception. Additionally, parents can actively participate by creating avatars together with their children, aiding them in exploring their identities in a supportive environment.

Additional research will be necessary to address the limitations of this study and expand understanding of the topic. First, while previous studies based on SRT have provided valuable insights with a comparable number of participants [71,73], the sample size of fifteen interviewees might have been insufficient to comprehensively understand the experiences of adolescent females in the SVW and their perspectives on avatar creation. Conducting additional interviews could provide a more in-depth understanding of the social representations held by users, particularly if the focus narrows down to a more specific age group within adolescence, given the rapid changes that often occur during this developmental stage.

Second, this research focused on Zepeto to the exclusion of other SVW platforms. Each of these platforms comes with its unique features and strengths. For instance, one of our interviewees illustrated, “While Zepeto is better for designing avatars, Roblox is better for playing games”. Ducheneaut et al. [36] have also indicated that each virtual world attracts a different user population, necessitating diverse customization systems to meet varying needs. In future studies, it would be valuable to compare perceptions of avatar creation across various platforms.

Third, while this study addressed how adolescents’ perception of their own identities influenced their avatar creation, it did not examine the potential impact of avatar creation on adolescents’ real-life self-perception. The finding that adolescent females often modify or abandon aspects of their actual identity traits to create an idealized version of themselves in avatars raises an intriguing question about whether the gap between actual self and virtual avatar might enhance or harm their self-esteem in real life. Particularly since adolescents are actively developing their sense of self, understanding the psychological impacts of their engagement with avatars can be a valuable area for future research.

Similarly, users’ attachment to or identification with their avatars could also be a significant variable to consider. Legal or criminal issues now arising out of the SVWs involve questions about the extent to which the experiences in SVWs can impact a user’s life. Specifically, questions are raised regarding whether instances of violence (e.g., sexual harassment) occurring between avatars in the virtual world can have detrimental effects on real-life users to a degree to which legal intervention may be justified or necessary. In this sense, exploring the intricate relationship between avatars and real selves becomes an urgent and practical area of research.

## Figures and Tables

**Table 1:** Benefits and challenges of online self-presentation for adolescent female users.

	Benefits	Challenges
Self-expression and interaction	- Creative self-expression - Enhanced self-image control - Validation through likes/follows	- Editing photos to conform to idealized beauty standards - Peer comparison
Relationships and friendships	- Expanded social circles - Enhanced emotional disclosure, quality friendships	- Cyberbullying - Online harassment - Unwanted sexual conversations
Identity exploration	- Exploring interests with like-minded individuals - Experimenting with virtual personas	- Persistent gender norms - Negative body image and dissatisfaction with actual self

**Table 2:** Examples of virtual world services popular among adolescents.

	Fortnite	Minecraft	Roblox	Zepeto	Avakin Life
Release year	2017	2011	2006	2018	2013
Publisher (Country of origin)	Epic Games (USA)	Mojang Studios (Sweden, now part of Microsoft)	Roblox Corporation (USA)	Naver Z Corporation (South Korea)	Lockwood Publishing (United Kingdom)
Number of users (as of 2023)	Over 500 million registered users (Over 231 million monthly active users)	Over 168 million monthly active users	Over 206 million monthly active users	Over 400 million registered users (Over 20 million monthly active users as of 2022)	Over 200 million registered users
Major characteristics	- Three different modes: cooperative game, battle royale game, creative mode - Custom skins, paid emote options, outfits, gears - Creative mode for designing structures and games on own island and inviting others	- Two different modes: creative mode and survival mode - Blocky sandbox for resource gathering and world building without predefined goals - Large player community to share maps and creations	- User-generated content platform for programming and playing games (Experiences) - Game development tools based on own scripting language, Lua - User sales of avatar accessories, creator models, and plugins on Marketplace	- Avatar customization with a focus on fashion and style - Advanced customization feature, Custom Pro, with extra fee - Sales of user-generated avatar templates and items - Virtual photo/video shoots, SNS-like posting and livestreaming features - In-app events and activities for users (e.g., K-pop fansign event)	- Customization and decoration of homes as well as avatars - Publisher -supplied items only - Random customization function for quick avatar creation - Designed with emphasis on social components over gaming - Social events such as parties and fashion contests

**Table 3:** Interviewee information.

Characteristics		N
Age	9	1
	10	2
	11	4
	13	3
	14	4
	15	1
Usage duration	Less than 1 year	5
	1–2 years	6
	More than 2 years	4
Frequency of use	1–2 times a week	1
	2–3 times a week	1
	3–4 times a week	2
	Almost daily	11
Daily usage time	Less than 1 h	3
	1–2 h	6
	More than 2 h	6
Time spent for avatar design	Less than 1 h	9
	1–2 h	3
	More than 2 h	2
	Cannot remember	1
Money spent for avatar design	None	7
	Less than 10,000 KRW ($7)	2
	10,000 KRW ($7)	1
	20,000 KRW ($15)	2
	45,000–300,000 KRW ($33–$222)	3
Experience of using other SVWs	None	5
	Roblox	10

**Table 4:** Topics related to adolescent females’ avatar creation in SVW.

No.	Topic	Example
T01	Replication of actual self	I’d like my avatar to look just like me. (Interviewee 2)
T02	Creation of new self	I’d like my avatar to look different from how I am in real life. (Interviewee 14)
T03	Ideal body	I’m pretty small and skinny in real life right now, so I want my Zepeto avatar to be tall and kinda big. (Interviewee 4) I want to make my avatar’s skin lighter than my actual skin because I think it looks prettier. (Interviewee 5)
T04	Identical gender	I feel more comfortable when I keep my avatar’s gender the same as my real gender. (Interviewee 8)
T05	Different gender	Since I’m a girl, I’d like to experiment with a male gender for my avatar, something I can’t experience in real life. (Interviewee 13)
T06	Student identity	Since I’m content with being a student in real life, I think a student avatar suits me best in Zepeto too. (Interviewee 2)
T07	Idealization of adults	I want to set my avatar’s age in the 20s because it seems like people in their 20s enjoy more freedom in life. (Interviewee 3)
T08	Autonomy of avatar creation	It’s an advantage of the metaverse that I can design my avatar just the way I like. (Interviewee 1)
T09	Surreal experience	For me, Zepeto is a world where I can experience things that I can’t do in real life. (Interviewee 13)
T10	Escapism	In Zepeto, there’s no school or private academy stressing me out. (Interviewee 7)
T11	Enjoyment	Zepeto is a fun world where something different happens every day, unlike my real life. (Interviewee 11)
T12	Virtual community	Zepeto feels like a SNS when I upload photos of my avatar dancing to my feed. (Interviewee 5) Zepeto lets me quickly connect with strangers and chat with people of all ages. (Interviewee 11)
T13	Financial barriers	I couldn’t buy the items I wanted because I didn’t have enough pocket money. (Interviewee 13) If it were easier to earn ZEMs, I could decorate my avatar exactly how I want, and make it look even more beautiful. (Interviewee 6)
T14	Imitation of reality	It feels just like reality when I hang out with friends in a school cafeteria in Zepeto. (Interviewee 2) In Zepeto, you can find plenty of realistic fashion items for decorating avatars. (Interviewee 8)
T15	Fantasy world	Zepeto is pure fantasy, not reality. (Interviewee 3) Zepeto is like an imaginary space that’s separate from reality. (Interviewee 4)
T16	Game-like service	Metaverse is a casual virtual space that gives off feelings like playing games. (Interviewee 1) Zepeto is the funniest game among many other games. (Interviewee 5)
T17	Differentiation from general games	I think Zepeto is different from typical games because you can customize it in so much detail. (Interviewee 4)

**Table 5:** Core-periphery structure.

No.	Topic	Coreness	Membership
T01	Replication of actual self	0.553	Core
T02	Creation of new self	0.439	
T06	Student identity	0.383	
T03	Ideal body	0.361	
T04	Identical gender	0.292	
T08	Autonomy of avatar creation	0.238	
T15	Fantasy world	0.136	Periphery
T12	Virtual community	0.132	
T09	Surreal experience	0.132	
T11	Enjoyment	0.106	
T17	Differentiation from general games	0.104	
T16	Game-like service	0.053	
T13	Financial barriers	0.047	
T14	Imitation of reality	0.044	
T05	Different gender	0.023	
T10	Escapism	0.012	
T07	Idealization of adults	0.006	

## Data Availability

The original contributions presented in this study are included in this article. Further inquiries can be directed to the corresponding author/s.
